# A responsive governance path to health equity: the role of state-led public interest litigation in China

**DOI:** 10.3389/fpubh.2025.1701396

**Published:** 2025-11-11

**Authors:** Fei Qi, Bin Yu

**Affiliations:** Law School, Hainan University, Haikou, China

**Keywords:** public interest litigation (PIL), health equity, responsive governance, China model, comparative law

## Abstract

Against the backdrop of the “Healthy China 2030” strategy, this paper examines China's unique Public Interest Litigation (PIL) system as an emerging and critical mechanism for safeguarding the health rights of vulnerable populations. The central thesis of this paper is that China's PIL should be understood not as a conventional rights-remedy instrument, but as a state-led innovation in “responsive governance.” This system, with the public procuratorate as its core actor, establishes an internal feedback loop designed to identify and rectify administrative regulatory failures, primarily through its pre-litigation procedures. The research finds that this system protects health rights through two distinct pathways: first, by providing universal, indirect protection through the regulation of social determinants of health, such as environmental quality and food safety; and second, by offering targeted, direct protection for specific groups, addressing issues like occupational health for migrant workers and accessibility of services for persons with disabilities. Through a systematic comparative analysis with the models of India (society-driven mobilization), South Africa (constitutional adjudication), and Brazil (individual rights realization), this paper illuminates the distinctiveness of the Chinese model. Its objective is focused on procedural administrative correction and enhancing governance efficacy, rather than on fundamental policy challenges. Although constrained by factors such as state-led agenda-setting, the model's emphasis on collective interests and systemic risks may generate more broadly shared public health benefits. This analysis provides a unique institutional case study on enhancing state governance capacity in the public health domain and contributes a nuanced perspective to global discussions on law, governance, and health equity.

## 1 Introduction

China's “Healthy China 2030” plan has elevated national health to a strategic priority, with the promotion of health equity as one of its central objectives (1). This national strategy explicitly aims to progressively narrow the disparities in health outcomes among different regions, urban and rural areas, and social groups. However, amidst rapid socioeconomic transformation, vulnerable populations—including migrant workers, persons with disabilities, the older adult(s), and women and children—continue to face significant barriers to the full realization of their right to health (2).

The right to health is a comprehensive concept, extending beyond timely healthcare to encompass the underlying social determinants of wellbeing, such as safe food, clean water, and healthy occupational and environmental conditions (3). This broad scope means the obstacles vulnerable groups face are not merely confined to disparities in access to medical services but are more deeply embedded in systemic risks. These risks, which include unsafe working conditions, disproportionate exposure to environmental pollution, and a lack of inclusive design in health-related products, stem from these very social determinants (3). Such systemic threats directly challenge the efficacy of the national public health governance system, making the translation of the macro-level principle of health equity into tangible safeguards for these specific groups a core governance challenge in implementing the “Healthy China” strategy.

To address this core governance challenge, China has increasingly utilized its unique system of Public Interest Litigation (PIL). A defining feature of this system is its authorization of the People's Procuratorate, the state's legal supervision organ, to initiate litigation when public interests are harmed (4). This design elevates the mechanism beyond mere judicial oversight, transforming it into an institutional instrument with dual functions of supervision and collaborative governance (5). In recent years, this legal tool, though not originally designed specifically for public health, has been innovatively applied to address institutional gaps in public health governance, emerging as a key mechanism for protecting the health rights of vulnerable populations (6).

Within the global landscape, China's approach is markedly distinctive. Several paradigms of public health litigation have emerged internationally. The first, exemplified by India, is a civil society-driven model. By relaxing the rules of legal standing, this model permits any public-spirited individual or organization to file lawsuits on behalf of marginalized groups unable to seek legal remedies themselves, thereby turning PIL into a bottom-up tool for social mobilization and health policy advocacy (7, 8). The second, represented by South Africa, is a constitutional rights adjudication model. This approach empowers constitutional courts to conduct reasonableness reviews of government health policies, enabling them to directly influence national health resource allocation or policy priorities by declaring existing policies unconstitutional (9, 10). A third paradigm, prominent in Brazil, can be described as an individual rights realization model. Here, citizens directly invoke the constitutional provision that “health is the right of all and the duty of the state” to file a large volume of individual lawsuits demanding that the state provide expensive medications or treatments to secure their personal health (11, 12).

China's PIL model diverges from all three. It is not driven by civil society (unlike India), it does not revolve around substantive review of policies based on constitutional rights (unlike South Africa), and it is not focused on individualized claims for remedies (unlike Brazil) (13, 14). This clear divergence gives rise to the central research questions of this paper. First, through which primary pathways does China's PIL system influence public health and the realization of health rights? Second, how does this mechanism exhibit differentiated protection patterns tailored to the specific circumstances of various vulnerable groups? Third, what structural factors constrain its potential?

The core argument of this paper is that China's PIL is not a traditional judicial lawsuit functioning as a rights-remedy tool. Instead, it should be conceptualized as an institutional innovation in “responsive governance”—defined here as an internal, state-led feedback mechanism that identifies and corrects regulatory failures within the administrative system to enhance governance efficacy without fundamentally altering existing power structures. This framing allows for a nuanced analysis of the mechanism's functions and limitations using neutral, technocratic language that aligns with the state's own goals of improving governance capacity and efficiency. To substantiate this thesis, this paper will first analyze the institutional architecture of China's PIL system. It will then, through case analysis, examine the dual pathways through which it impacts public health and health rights. Building on this, the paper will explore the systemic constraints facing the mechanism. Finally, through a systematic comparison with the models in India, South Africa, and Brazil, it will reveal the unique logic and theoretical implications of the Chinese model, concluding with policy recommendations for its future development.

## 2 The institutional architecture of China's public interest litigation: a state-led governance instrument

China's PIL system is not the product of a single legislative act but has undergone a rapid and systematic evolution. Following regional pilots initiated in 2015, the system was formally established nationwide in 2017 through amendments to the *Civil Procedure Law* and the *Administrative Litigation Law*. Its statutory scope has also expanded dynamically. From an initial focus on four core areas—ecological environment and resource protection, food and drug safety, protection of state-owned property, and transfer of state-owned land use rights—it has grown to encompass new fields such as the protection of minors, personal information, and consumer rights, forming a dynamic “4+N” jurisdictional framework.

### 2.1 The centrality of the procuratorate as the core actor

The most defining characteristic of this institutional architecture is the central role of the People's Procuratorate as the primary plaintiff (15). This stands in stark contrast to models like India's, where non-governmental organizations (NGOs), social activists, or private individuals are the main litigants (7, 8). As the state's designated legal supervision organ, the procuratorate is vested with statutory powers of investigation and verification and can effectively coordinate with other administrative departments (16). This enables it to overcome the structural barriers that often plague individual or civil society litigants in other countries, such as insufficient funding, limited investigative capacity, and administrative resistance.

This unique positioning as a state organ grants the procuratorate a structural advantage in legal contests with local government departments or large corporations, significantly enhancing the success rate and deterrent effect of the litigation. Empirical data confirms this dominance, showing that the number of cases initiated by procuratorates far exceeds those brought by social organizations. This dual identity—as both a state prosecutor and a supervisor of the administration—is fundamental to the system's operation (5). It allows the state to address public grievances and administrative failures in a controlled manner, thereby enhancing its governance capacity without opening the door to unpredictable legal challenges that could undermine the governing order.

### 2.2 The “pre-litigation procedure” as a governance tool

The governance function of the system is most clearly embodied in the “pre-litigation procedure” of administrative PIL. According to the law, before formally filing an administrative lawsuit in court, the procuratorate must first issue a “prosecutorial recommendation” to the administrative agency suspected of improper performance of its duties (17). This recommendation urges the agency to correct its illegal actions or fulfill its statutory responsibilities within a prescribed period. Only if the agency fails to undertake effective rectification after receiving the recommendation will the procuratorate initiate formal litigation.

This design is not merely a procedural prerequisite; it is the core of the system's governance philosophy. It effectively transforms a potentially adversarial judicial conflict into an internal, consultative, and corrective process. The primary objective is not to punish or negate the administrative agency through a court judgment but to use the pressure of procuratorial supervision—an “intra-system” check—to incentivize the administrative system to engage in self-repair. This mechanism prioritizes internal coordination and administrative efficiency, seeking to resolve problems while preserving the authority of the administrative agency and avoiding open, intense confrontations (18–20). This contrasts sharply with litigation models where a lawsuit is often a citizen's last resort in a public confrontation with the state.

The widespread use of prosecutorial recommendations demonstrates that, in practice, China's PIL functions more as an instrument of collaborative governance and administrative supervision than as a purely judicial adjudication tool (21, 22). Its purpose is to perfect, rather than challenge, the administrative functions of government agencies. This managed and negotiated approach to justice, conducted primarily within the state apparatus before reaching a public courtroom, reveals the system's nature as a mechanism for controlled conflict resolution. It functions as an internal corrective mechanism as much as a legal instrument, allowing the state to address genuine public concerns and systemic risks in a way that reinforces, rather than threatens, its overall stability and governance.

## 3 Practical pathways and limitations in safeguarding health rights

In practice, China's PIL system has forged two distinct pathways for safeguarding citizens' health rights within the public health domain. One pathway provides universal, indirect protection by improving the social and environmental determinants of health. The other offers targeted, direct protection by addressing the specific challenges faced by particular vulnerable groups.

### 3.1 The indirect pathway: regulating social determinants of health

The most extensive application of PIL in the health sector has been improving key social determinants of health (23, 24). This pathway is particularly prominent in litigation concerning environmental protection and food and drug safety. While the immediate goal of such litigation is to protect a general public interest, the resulting mitigation of systemic risks has a disproportionately significant positive impact on groups made vulnerable by their socioeconomic status or physiological condition (25, 26).

In the environmental sphere, PIL has become a core mechanism for addressing environmental pollution, a critical threat to public health (27). Environmental pollutants pose direct health risks to communities near their source, especially children and older people, who are more physiologically susceptible. Environmental pollutants pose direct health risks to communities near their source, especially children and older people, who are more physiologically susceptible. Empirical studies demonstrate that within these vulnerable populations, environmental PIL generates differentiated health benefits based on socioeconomic status. A tracking study in Hubei Province found that PIL-driven pollution control reduced respiratory disease incidence among children from low-income families by 32%, compared to 18% among children from high-income families (28).

Since 2019, China's procuratorates have initiated 336,000 environmental PIL cases, with an increasing focus on resolving systemic and trans-regional ecological problems (29). The comprehensive management of the Yangtze River Basin serves as a prime example. In response to complex, long-standing issues such as industrial effluent discharge, illegal sand mining, and agricultural non-point source pollution, procuratorates have used PIL to deeply engage with and promote the effective implementation of the *Yangtze River Protection Law* (30). Judicial precedents show that these lawsuits not only hold polluters civilly liable but, more importantly, compel local governments—through pre-litigation recommendations and court orders—to fulfill their statutory duties of ecological restoration and long-term regulatory oversight. The public health co-benefits of such large-scale environmental governance are substantial. Improving water quality directly reduces the risk of water pollution-related diseases for hundreds of millions of residents along the river, including many vulnerable individuals in rural and underdeveloped areas.

Similarly, in food and drug safety, PIL provides universal health protection by correcting systemic risks within supply chains. Recent data illustrate the sustained intensity of this supervisory function: in 2024 alone, procuratorates nationwide filed 26,000 PIL cases concerning food and drug safety and other consumer rights protection matters. The supervisory scope has expanded from traditional offline markets to safety loopholes in new business models like community group buying and livestream e-commerce. For instance, in response to the problem of excessive pesticide residues in agricultural products, China's procuratorial intervention has prompted agricultural and market supervision authorities to strengthen source control and market inspections. In guiding cases issued by China's highest judicial organs, procuratorates have not only demanded compensation from producers who use banned or restricted pesticides but have also successfully compelled local governments to establish and improve traceability systems for agricultural product quality and safety (31). These cases transcend individual punishment, establishing stricter industry standards and regulatory responsibilities through judicial decisions. The protective effects benefit all consumers, but this institutional safeguard is crucial for those who are more vulnerable due to their socioeconomic status or physiological condition.

### 3.2 The direct pathway: targeted protection for specific vulnerable groups

Beyond providing universal protection, PIL is increasingly being used to identify and intervene in the specific health challenges faced by particular vulnerable groups (25). This functional evolution signifies a shift from addressing general public health risks to actively promoting health equity for specific populations.

The protection of occupational health for migrant workers is a significant application of this targeted intervention. Due to their precarious employment status and difficulties in providing evidence, this group often faces systemic barriers when seeking individual remedies, especially concerning risks of occupational diseases like pneumoconiosis. PIL offers a mechanism for systemic intervention. Procuratorates can initiate supervision over regulatory gaps, such as an employer's failure to report occupational hazards, provide health examinations, or supply compliant protective equipment (32). For example, in cases involving enterprises with excessive noise levels that caused long-term harm to the occupational health of migrant workers, prosecutorial recommendations led not only to penalties and rectifications for the non-compliant companies but also to the establishment of a long-term regulatory framework for the sector. Such interventions directly address critical points of failure that harm workers' right to health.

This targeted pathway also extends to enhancing the accessibility of health services and products for persons with disabilities and the older adult(s) (33). Litigation has been used to address systemic barriers in both the physical and informational environments. A notable area of intervention has been in removing accessibility barriers to emergency services. In response to the inability of individuals with hearing and speech impairments to effectively use emergency hotlines (120/119), procuratorates in multiple localities have used PIL to push for system upgrades, resulting in the addition of text-based emergency reporting functions (34). This technological modification effectively removes a critical obstacle to the equal right to access emergency medical assistance. Likewise, addressing the medication risks faced by older adult(s) patients due to the small font size on drug instruction labels, litigation has prompted regulatory agencies to develop pilot programs for “elder-friendly” designs, including the promotion of large-print versions and QR codes for audio-assisted reading, thereby reducing the risk of information misinterpretation (34). However, in the realm of accessibility infrastructure development, while technical improvements to emergency service systems have benefited all individuals with special needs, the actual effectiveness of usage remains influenced by factors such as digital literacy and device accessibility. This suggests that even direct protective measures targeting specific groups may still be affected by intra-group differences in their implementation and outcomes (35).

The specific health risks faced by women and children have also become a focus of procuratorial supervision, with judicial practice touching upon several concrete areas. For instance, in the medical aesthetics industry, which has a high concentration of female consumers, several procuratorates have used PIL to address issues like unlicensed practice and the use of counterfeit or substandard products that threaten consumer health (36). These actions successfully prompted joint enforcement campaigns by health and market supervision authorities, leading to improved industry standards. In the field of minor protection, a series of PIL cases, based on considerations of the physiological, psychological, and sociocultural risks that tattooing poses to minors (37), has contributed to the issuance of a national policy prohibiting businesses from providing tattoo services to minors.

The following table (Table 1) summarizes these two pathways, illustrating the dynamic development of the PIL system in promoting health equity. However, the analysis of these cases also reveals that the effectiveness of both indirect and direct pathways is subject to several common and deep-seated constraints.

**Table 1:** A Typology of pathways for safeguarding the health rights of vulnerable groups via PIL in China.

**Institutional pathway**	**Primary vulnerable groups affected**	**Legal basis**	**Typical case example**	**Nature of health right safeguarded**
Indirect pathway: environmental protection	General residents in affected areas, particularly physiologically vulnerable children and the older adult(s)	*Environmental Protection Law*; *Civil Procedure Law*	A procuratorate sues a chemical company for illegally discharging wastewater into a river	The right to a healthy and safe living environment; prevention of pollution-related diseases
Indirect pathway: food and drug safety	All consumers, particularly physiologically vulnerable children and the older adult(s)	*Food Safety Law*; *Consumer Rights Protection Law*; *Civil Procedure Law*	A procuratorate sues the manufacturer of non-compliant infant formula or the seller of expired pharmaceuticals	The right to safe food and medicine; prevention of poisoning and adverse health outcomes
Direct pathway: occupational health	Migrant workers	*Law on Prevention and Control of Occupational Diseases*; *Labor Law*	A procuratorate issues a recommendation to a factory for failing to provide dust masks and conduct health checks for pneumoconiosis risk	The right to safe and healthy working conditions; prevention of occupational diseases
Direct Pathway: Accessible Environments and Services	Persons with disabilities, the older adult(s), and others with accessibility needs	*Law on the Protection of Persons with Disabilities*; *Law on the Creation of an Accessible Environment*	Compelling the modification of the “120” emergency hotline to serve the hearing-impaired; promoting “elder-friendly” redesign of drug labels	The right to non-discriminatory access to essential medical information and emergency services
Direct pathway: intervention in specific group health risks	Women, children, the older adult(s), and other vulnerable groups	*Drug Administration Law*; *Consumer Rights Protection Law*	Litigation against the use of counterfeit products in the medical aesthetics industry or the fraudulent sale of health products to the older adult(s)	The right to bodily integrity and safety in consumer and medical environments

### 3.3 Systemic constraints within the state-led framework

Despite its achievements, the full potential of China's PIL system is constrained by several systemic factors. While these challenges are not unique to China, they manifest in specific ways within its state-led framework.

First, at the stage of case selection and initiation, the ambiguity of legal application is a primary constraint. Although the “4+N” jurisdictional framework is open-ended, the law does not provide a clear definition of what constitutes a “public interest” in the context of emerging or cross-sectoral health rights issues (38). This leads procuratorates in practice to favor traditional cases with a clearer legal basis and less social controversy, such as typical environmental pollution or food safety violations. For more profound health equity issues involving complex medical ethics, health resource allocation, or systemic discrimination, procuratorates exhibit varying degrees of caution. This judicial prudence reflects the inherent logic of the system as a state governance tool: its primary task is to ensure the stable enforcement of the existing legal framework, not to pioneer new legal territory, which is considered the purview of the legislature (39).

Second, even when a case is initiated, obtaining scientific evidence often becomes a critical bottleneck during the litigation process. In complex environmental and public health lawsuits, scientific evidence is decisive (40). However, securing authoritative and neutral “judicial appraisal” opinions is difficult, mainly due to high costs and a scarcity of qualified appraisal resources. This challenge is globally prevalent; in the U.S. judicial system, for example, litigants must invest enormous resources in expert evidence battles under the “Daubert standard,” where resource asymmetry can affect judicial fairness (41). China's dilemma lies in its still-developing market for appraisal services (42), coupled with the budgetary constraints that procuratorates, as state agencies, face in allocating public funds for expensive expert fees (43). This challenge, however, also presents an opportunity for institutional innovation. A state-led litigation model is, in theory, better positioned than a private litigation model to address this market failure. Indeed, procuratorates in many provinces are exploring collaborative approaches to introduce external expert resources to support public appraisal needs (44).

Finally, at the end of the litigation process, the effective enforcement of judgments presents a “last mile” challenge. Similar to judicial practice in many countries, difficulty in enforcement is a key obstacle preventing China's PIL from realizing its full social value. Even if the procuratorate wins a lawsuit, the environmental remediation, damage compensation, or institutional improvements mandated by the judgment may not be fully implemented due to local protectionism, inter-departmental conflicts of interest, or prohibitive enforcement costs (21, 45). This problem is particularly acute when a judgment affects core local economic interests or powerful corporations. To address this, Chinese procuratorates are exploring ways to strengthen their post-judgment supervisory functions, using follow-up actions and further prosecutorial recommendations to ensure compliance (46). This, to some extent, supplements traditional judicial enforcement mechanisms and once again highlights the unique role of the procuratorate as a cross-departmental coordinator and supervisor within this model (47).

## 4 The Chinese model in a global context: a comparative discussion

**Figure 1:**
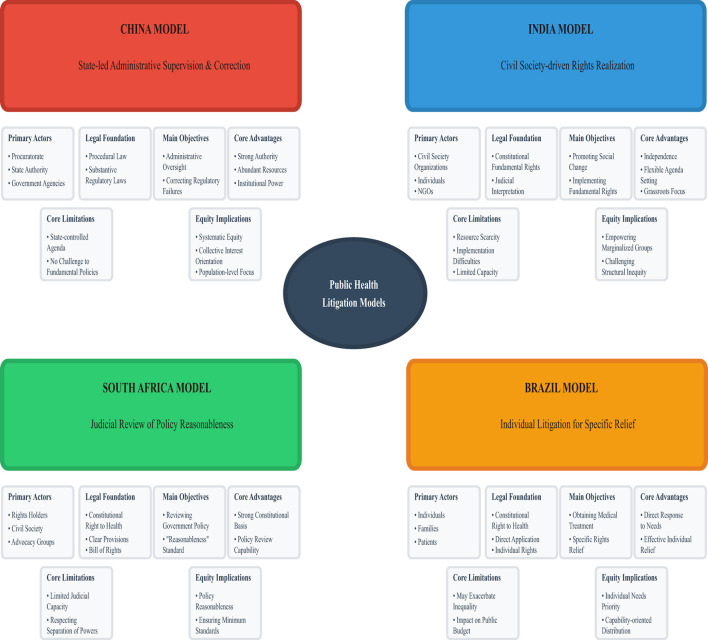
Comparative models of public health litigation.

Placing China's PIL model in a global comparative perspective enables a deeper understanding of its institutional logic, inherent trade-offs, and theoretical implications. This paper selects India, South Africa, and Brazil as comparative cases based on the following considerations: representativeness—these three countries respectively represent the three main institutional types of public health litigation in the contemporary global context; contractiveness—they form stark contrasts with the Chinese model across core dimensions including the source of power, litigation objectives, and protective effects; typicality—the institutional practices of these countries have significant influence in their respective regions, providing important reference points for understanding different developmental paths; comparability—like China, these are all developing nations that face similar challenges in realizing health rights, including significant health inequalities, resource constraints, and the need to balance economic development with public health priorities. This shared developmental context makes their different institutional responses to health equity challenges particularly illuminating for comparative analysis (Figure 1).

### 4.1 State-led power vs. society-driven mobilization (China vs. India)

The institutional logic of China's PIL model stands in sharp contrast to India's archetypal society-driven model. While China's state-led approach effectively addresses the challenges of resources, enforcement, and stability often faced by society-driven models, the two systems reflect different value orientations in their agenda-setting and operational modes.

India's model is quintessentially society-driven, deriving its legitimacy and dynamism from the interplay between the judiciary and an active civil society. Social organizations, activists, and public intellectuals are the core forces identifying problems and initiating litigation, while courts, through judicial activism, transform statutory rights into instruments of change (7, 8). The strength of this model lies in its independence and flexibility, enabling it to raise challenging issues from the grassroots. However, it also faces inherent operational tensions, including severe resource constraints, the potential for litigation abuse, and difficulties in enforcing judgments (8).

In contrast, the “dual-track” design of China's PIL system has, in practice, followed a clear evolutionary path. Although the law provides a space for social organizations to participate in litigation, their effectiveness is severely limited by structural barriers, including high standing requirements, a chronic lack of resources, and, most critically (48), the absence of statutory investigative powers (49). As a result, the number of cases initiated by social organizations has remained at a relatively low level. This structural weakness of the civil society track has objectively created the conditions for the strengthening and functional substitution of the state-led model. The state-empowered procuratorate, leveraging its formidable investigative authority, sufficient financial resources, and political clout, can systematically overcome these barriers. It demonstrates an efficacy and success rate in handling complex cases involving local governments or large corporations that social organizations cannot match. Therefore, the institutional landscape of Chinese PIL did not emerge from a sudden top-down design but evolved through a process where the functional deficits of social organizations were met by the enhanced capacity of the procuratorate. This evolution reflects the system's intrinsic functional priority: it favors an internal supervisory path led by a state organ, which offers high certainty and strong enforcement, over an external mobilization path that relies on social spontaneity and is relatively less efficient. This has firmly established the absolute dominance of the procuratorate.

China's state-led model demonstrates superior procedural efficiency through rapid resolution timelines (50), systematic protection of vulnerable populations (51, 52), and effective resource utilization in administrative correction (53). Conversely, India's society-driven PIL system suffers from severe operational inefficiencies, with cases often experiencing protracted timelines and prolonged resolution processes that can extend across multiple years or even decades (54). The Indian PIL system faces inherent structural problems including chronic resource constraints among civil society organizations, difficulties in case enforcement, and the potential for litigation abuse. Moreover, empirical studies from the 2000s reveal a troubling evolution: Indian PIL cases increasingly favor advantaged individuals over the poor, with quantitative analysis showing that the system now systematically benefits middle and upper classes rather than the weaker sections of society it was originally designed to protect (54). The Indian model's dependence on judicial discretion and informal procedures, while providing flexibility, has resulted in inconsistent outcomes and raised concerns about the separation of powers as courts increasingly assume legislative and executive functions (54).

However, the limitations of this state-led model are equally clear. The litigation agenda is set entirely by the procuratorate, and its scope and intensity are constrained by overall national priorities. This makes the system effective at correcting specific administrative illegalities but ill-suited for debating macro-level health policies or addressing structural problems linked to core economic interests.

### 4.2 Procedural administrative correction vs. substantive constitutional adjudication (China vs. South Africa)

The core difference between the Chinese and South African models lies in the fundamental objective (*telos*) of litigation—that is, the depth and manner in which judicial power intervenes in public affairs.

South Africa's model is a classic rights adjudication model that positions judicial power as a strong external check on executive power. Based on the constitutional right to health, its core function is to authorize courts to conduct substantive reasonableness reviews of government health policies (55). This means courts are empowered to directly engage with the content of policy, assessing whether government measures are “reasonable.” However, this powerful form of judicial review has also mired the system in continuous controversy. Critics argue that when court rulings directly affect national budget allocations and policy priorities, judicial power risks encroaching upon the core domains of the legislative and executive branches, sparking intense debates about the separation of powers and the appropriate role of the judiciary (10, 56). In short, while the South African model provides robust protection for citizens' constitutional rights, it must also confront accusations of “judicial overreach.”

China's PIL follows a different path, with a more restrained and procedural objective, and its institutional design deliberately avoids similar direct conflicts between the judiciary and the executive. It primarily focuses on legality control—supervising whether administrative agencies have complied with existing laws and fulfilled their statutory duties—while generally not touching upon the reasonableness of legislation or policy itself (57). In PIL, the procuratorate's function is positioned as that of a collaborative governance tool aimed at perfecting the internal operations of the administrative machinery (58). Its purpose is to supervise and repair from within, respecting the authority of the executive branch, rather than making external value judgments.

Based on our comparison of the effectiveness between China and South Africa, the choice between these two models reflects the different governance choices between transformative depth and governance stability, reflecting two distinct governance philosophies and risk calculations. The South African model emphasizes transformative depth. To ensure the ultimate realization of constitutional rights, it accepts the risk of “judicial encroachment on the executive” by empowering courts to conduct substantive reviews of policy merits. Its constitutional rulings demonstrate a unique capacity for paradigmatic interventions in the health policy domain, with significant Constitutional Court judgments directly driving government adjustments to national housing policies and HIV treatment policies, providing “breakthrough” substantive rights protection for specific vulnerable groups (59–61). The Chinese model, in contrast, emphasizes governance stability and administrative system coordination, prioritizes the autonomy of the administrative system and the overall efficiency of national governance. It embeds procuratorial supervision within the governance system, using internal consultation and procedural correction to ensure administrative compliance, thereby avoiding direct confrontation between different branches of state power. This model demonstrates excellent administrative efficiency, excelling at addressing specific regulatory failures—ensuring compliance with existing environmental standards, correcting administrative oversights, and improving the implementation of established policies—achieving broader and more systematic improvements in environmental and consumer protection that effectively benefit the general public (62–65). Both models have their respective institutional advantages and applicable scenarios, reflecting the rational choices made by different countries based on their governance traditions and practical needs.

### 4.3 Public goods and the “equity paradox” of distributive effects (China vs. Brazil)

The comparison between China and Brazil uncovers a profound paradox regarding the distributive effects of health litigation. While both nations employ judicial pathways to address health rights, their differing models yield starkly contrasting outcomes for health equity.

Brazil's health litigation model is rooted in its broad constitutional right to health but, in practice, manifests as the realization of individualized claims. The typical pathway involves citizens suing the state to obtain specific, often expensive, medications or treatments (66). The advantages of this model lie in its ability to provide strong rights protection for individuals, particularly in forcing systematic adjustments when facing government inaction or improper resource allocation. However, extensive research confirms that its beneficiaries are disproportionately middle- and upper-class individuals with better access to legal resources (67, 68). This can, in turn, lead to public health budgets being used to satisfy the special needs of a few, objectively exacerbating systemic health inequalities. The outcome of such litigation is, in essence, an excludable “private good.”

In stark contrast, China's Public Interest Litigation follows a distinctly collectivist and system-oriented logic. Its subject matter is not an individualized interest but an indivisible “public interest”—whether that involves remediating a polluted river or regulating an entire industry's safety standards. Consequently, the results of such litigation inherently constitute “Public goods”—defined as non-excludable public benefits that cannot be reserved for specific individuals but instead extend equally across entire communities or consumer groups (69). The most vulnerable populations, who lack the resources to pursue individual claims, are precisely those who receive equal protection from these outcomes.

The two models exhibit significant differences in depth, breadth, and mechanisms of health intervention. Brazil's individual rights model possesses powerful “breakthrough intervention” capabilities, defined as the capacity to generate transformative medical access where none previously existed, enabling courts to compel the government through judicial decisions to provide cutting-edge medical technologies for specific patient populations (70), establish treatment precedents that benefit entire disease categories, and drive fundamental adjustments to national health policies (71). This model demonstrates unique advantages in addressing highly specialized and resource-intensive medical needs, such as rare disease treatments and organ transplants, creating institutionalized solutions for special medical needs that are overlooked within conventional policy frameworks. In contrast, China's collective interest model demonstrates exceptional systematic efficiency in population-level health risk prevention and control, particularly in areas involving large-scale population health threats such as environmental pollution control and food safety regulation, achieving rapid and comprehensive risk mitigation through administrative correction. However, this model faces structural limitations when addressing health needs that require individualized treatment protocols or breakthroughs of existing policy frameworks, as its procedural characteristics make it difficult to handle complex health equity issues that necessitate fundamental resource reallocation or policy innovation (72). The two models serve different health equity dimensions: Brazil's model specializes in “vertical breakthrough”—creating treatment opportunities from scratch for specific populations, while China's model excels at “horizontal coverage”—providing foundational health protection for broad populations.

This comparison reveals an “equity paradox” in health litigation—a phenomenon whereby litigation models formally centered on individual constitutional rights (Brazil) may paradoxically generate inequitable distributive outcomes, while state-led collective litigation models not premised on individual rights claims (China) can produce more equitable public health outcomes. This suggests that on the path to health equity, there is no necessary linear relationship between how formally “rights-empowering” an institutional tool is and its substantive capacity to promote “distributive fairness.” Rather than suggesting the superiority of either model, this paradox highlights the need for more nuanced understanding of how different institutional designs navigate the complex trade-offs between individual rights protection, distributive effects, and governance stability in context-specific ways.

### 4.4 Synthesizing the Chinese model as responsive governance

A comprehensive review of these comparisons reveals that the uniqueness of the Chinese model lies not in any single feature but in the internal coherence of its various dimensions. Its state-led nature addresses the resource constraints of society-driven models; its procedural focus on administrative correction avoids the power-boundary disputes of substantive judicial review; and its collective interest orientation produces more equitable distributive effects than individual rights models. These interwoven features shape its core identity, which this paper conceptualizes as an instrument of “responsive governance.” However, this unique synthesis is not without its own internal tensions and practical challenges, which merit a closer examination of its operational logic and the scholarly critiques it has attracted.

An in-depth analysis of its internal logic and operational mechanisms reveals that the core of this “responsive governance” is not direct rights relief or policy reform, but rather the correction of administrative agencies' performance through procedural supervision. The achievement of this objective results from the specific configurations of litigant entities and the design of supervisory channels in “responsive governance”. While it incorporates multi-party participation mechanisms, including litigation by social organizations, in practice, state-led public interest litigation serves as the primary driver, supplemented by various supervisory channels such as public reporting and hearings. This supervisory framework, with the procuratorate at its core and multiple channels as supplements, ensures sustained and effective oversight of administrative agencies. Judicial practice data further confirms the system's supervisory orientation. In barrier-free public interest litigation, for example, a striking 99.61% of cases target administrative agencies as defendants (73), which clearly demonstrates that the system primarily supervises administrative regulatory bodies in fulfilling their statutory duties through litigation mechanisms, thereby safeguarding public interests.

Therefore, the essence of this “responsive governance” is to leverage litigation to compel self-correction by administrative agencies. Its core mechanism is not direct judicial adjudication on substantive matters or intervention in policymaking. Instead, it uses the external pressure of procuratorial supervision to activate an endogenous momentum for self-improvement within the existing governance framework. This institutional arrangement astutely achieves effective restraint on administrative inaction or misconduct while avoiding direct judicial intervention in executive power.

While this operational logic highlights the model's internal coherence, its principled design is also the source of its inherent boundaries and has attracted some scholarly critique. The system's strict adherence to legality review over reasonableness review is a core limitation, meaning the procuratorate's role is to supervise clear statutory violations, rather than scrutinize the substantive rationality of decisions made within an agency's legitimate discretion (74). This very design choice—prioritizing a dominant, state-led supervisory role—gives rise to deeper theoretical tensions, as some scholars question whether this model is fully compatible with the neutral and passive character of judicial power, the principle of prosecutorial restraint, and the structure of equal adversarial proceedings in civil litigation (75).

Operationally, further critiques arise from the system's practical application. Some analysts argue that the heavy emphasis on pre-litigation procedures risks diluting the system's judicial nature, transforming what should be a formal lawsuit into a “negotiation and mediation mechanism.” This “deviation” has led to calls for strengthening the procuratorate's supplementary, rather than primary, role and diversifying the range of eligible plaintiffs (76). Furthermore, empirical studies reveal a gap between specific institutional designs and their practical applications. For instance, the seven-person collegiate panels, designed to enhance public participation, are often not correctly formed, raising questions of formal legality. Even when they are, procedural flaws can render the lay members a “silent majority,” undermining the intended democratic function of the trial and questioning its substantive legitimacy (77).

This entire analysis clarifies the functional boundaries and calculated trade-offs of the system. It is not designed to serve as a platform for social confrontation, like the Indian model, a forum for constitutional adjudication, like the South African model, or a window for cashing in on individual rights, like the Brazilian model. Instead, it is an internal self-improvement mechanism. Its fundamental objective is to enhance governance capacity by addressing regulatory failures within the existing power framework, rather than serving as a catalyst for rights-based challenges to the framework itself. Understanding this calculated trade-off is crucial for moving beyond simplistic dichotomies and for accurately positioning the Chinese model within the global spectrum of public interest governance.

## 5 Limitations and future prospects

It must be candidly acknowledged that this study has significant limitations in terms of data sources. Due to the particular nature of the research topic, we primarily rely on government documents, legal texts, and official statistical data, lacking first-hand observations and assessments from diverse actors such as independent lawyers, civil society organizations, and affected communities. Such limitations in sources may lead to biases in our understanding of the system's actual operational effectiveness and social impact, particularly potentially underestimating the challenges it faces in practice or failing to fully reflect the genuine experiences of different stakeholders.

Based on the findings and limitations of this study, future research should deepen and expand our understanding of PIL mechanisms for health rights protection across multiple dimensions. First, there is an urgent need for more in-depth empirical research through field investigations, in-depth interviews, and participatory observation methods to systematically collect first-hand data from diverse stakeholders, including public interest lawyers, environmental organizations, beneficiary communities, and government agencies, in order to more comprehensively assess the actual operational effectiveness and social impact of the system. Second, more refined evaluation frameworks should be established to develop indicator systems capable of quantifying the degree of health equity improvement, particularly requiring the construction of measurement tools to capture differentiated benefits across various social groups. Third, comparative institutional research needs further deepening, not only expanding the scope of comparative cases to include experiences from more developing countries, but also strengthening tracking analysis of the dynamic processes of institutional change, exploring adaptive evolutionary mechanisms of PIL systems under different political and economic environments. Finally, as global governance challenges become increasingly complex, exploring the role of PIL in addressing transnational health threats, climate change health impacts, and other global issues will provide important institutional innovation experiences for building more inclusive and sustainable global health governance systems.

## 6 Conclusion

The analysis in this paper demonstrates that China's Public Interest Litigation is not a traditional rights-remedy mechanism but should be understood as an innovation in “responsive governance” aimed at enhancing state capacity. Through the dual pathways of “indirect universal protection” and “direct targeted intervention,” it has shown unique advantages and significant potential in promoting health equity. More importantly, through a systematic comparison with the models of India, South Africa, and Brazil, this paper has identified the three core pillars of the Chinese model: it uses a state-led position to overcome the resource bottlenecks of social mobilization; it employs procedural administrative correction to avoid the power-boundary disputes of substantive judicial review; and it pursues more universal distributive fairness than individual rights realization models by focusing on the collective public interest.

Of course, this institutional choice also entails clear functional boundaries and inherent trade-offs. As previously discussed, the model exchanges the flexibility of a society-driven agenda for the certainty of a state-led one; it trades the sharpness of external oversight for the efficiency of internal collaboration. This positioning determines that it can effectively correct specific administrative failures but is ill-equipped to address fundamental health policies or regional development models. From the perspective of the judiciary's functional role, the future evolution of this system will depend not on subverting its fundamental nature as an internal governance tool, but on making refinements within its existing framework. To this end, the analysis points to several key paths forward: clarifying the scope of health rights issues through legislative interpretation to provide authorization for intervening in more complex equity domains; leveraging the state's advantages to establish a public appraisal support system to overcome evidence bottlenecks; further strengthening post-judgment supervision and collaborative enforcement to ensure a closed governance loop; and exploring a combination of “state empowerment” and “social enablement” to moderately activate the supplementary role of social participation without weakening the dominant role of the state.

Based on the previous analysis, to enhance the effectiveness of public interest litigation in promoting health equity and achieve a balance between equity and efficiency, the following recommendations are proposed: first, in terms of legislation, clarify the scope of health-related public interests through detailed statutory provisions and judicial interpretations, define the boundaries between individual health rights and collective health interests, while providing explicit authorization for procuratorial intervention in complex health equity issues involving social determinants of health. Second, at the institutional level, procuratorates need to establish good communication with health administrative agencies and develop standardized procedures for health impact assessment to better identify and pursue cases with significant health equity implications, and by streamlining coordination mechanisms between procuratorates and health administrative agencies, establish stronger post-judgment monitoring systems to ensure compliance and measure health outcomes, as well as develop more direct pre-litigation procedural guidelines to maximize the corrective potential of procuratorial recommendations. Third, regarding participation mechanisms, while maintaining state leadership, substantive participation of social organizations—including public interest groups, professional associations, and community-based organizations—should be promoted, making them important actors in identifying health equity issues, providing technical expertise, and monitoring implementation outcomes.

Ultimately, the evolution of China's PIL will not only be a microcosm of the country's legal development but will also serve as a critical window through which to observe the degree to which the principle of equity is realized within the “Healthy China” strategy. It offers a unique and complex case study for global governance: how a state, without undertaking disruptive structural reforms, can use a state-led, controlled judicial instrument to enhance its governance capacity and respond to increasingly complex social challenges. The experiences, trade-offs, and lessons from its efforts to promote health equity will undoubtedly provide profound insights for public health governance worldwide.
